# Ruling out COVID-19 by chest CT at emergency admission when prevalence is low: the prospective, observational SCOUT study

**DOI:** 10.1186/s12931-020-01611-w

**Published:** 2021-01-12

**Authors:** Ulf Teichgräber, Amer Malouhi, Maja Ingwersen, Rotraud Neumann, Marina Reljic, Stefanie Deinhardt-Emmer, Bettina Löffler, Wilhelm Behringer, Jan-Christoph Lewejohann, Andreas Stallmach, Philipp Reuken

**Affiliations:** 1 Department of Radiology, Friedrich-Schiller-University, Jena University Hospital, Am Klinikum 1, 07747 Jena, Germany; 2 Department of Internal Medicine, Friedrich-Schiller-University, Jena University Hospital, Am Klinikum 1, 07747 Jena, Germany; 3 Institute of Medical Microbiology, Friedrich-Schiller-University, Jena University Hospital, Am Klinikum 1, 07747 Jena, Germany; 4 Department of Emergency Medicine, Friedrich-Schiller-University, Jena University Hospital, Am Klinikum 1, 07747 Jena, Germany

**Keywords:** COVID-19, Computed tomography, Prevalence, Reverse transcriptase polymerase chain reaction, Sensitivity and specificity, Severe acute respiratory syndrome coronavirus 2

## Abstract

**Background:**

It is essential to avoid admission of patients with undetected corona virus disease 2019 (COVID-19) to hospitals’ general wards. Even repeated negative reverse transcription polymerase chain reaction (RT-PCR) results do not rule-out COVID-19 with certainty. The study aimed to evaluate a rule-out strategy for COVID-19 using chest computed tomography (CT) in adults being admitted to the emergency department and suspected of COVID-19.

**Methods:**

In this prospective, single centre, diagnostic accuracy cohort study, consecutive adults (≥ 18 years) presenting with symptoms consistent with COVID-19 or previous contact to infected individuals, admitted to the emergency department and supposed to be referred to general ward were included in March and April 2020. All participants underwent low-dose chest CT. RT-PCR- and specific antibody tests were used as reference standard. Main outcome measures were sensitivity and specificity of chest CT. Predictive values were calculated based on the theorem of Bayes using Fagan’s nomogram.

**Results:**

Of 165 participants (56.4% male, 71 ± 16 years) included in the study, the diagnosis of COVID-19 was confirmed with RT-PCR and AB tests in 13 participants (prevalence 7.9%). Sensitivity and specificity of chest CT were 84.6% (95% confidence interval [CI], 54.6–98.1) and 94.7% (95% CI, 89.9–97.7), respectively. Positive and negative likelihood ratio of chest CT were 16.1 (95% CI, 7.9–32.8) and 0.16 (95% CI, 0.05–0.58) and positive and negative predictive value were 57.9% (95% CI, 40.3–73.7) and 98.6% (95% CI, 95.3–99.6), respectively.

**Conclusion:**

At a low prevalence of COVID-19, chest CT could be used as a complement to repeated RT-PCR testing for early COVID-19 exclusion in adults with suspected infection before referral to hospital’s general wards.

*Trial registration* ClinicalTrials.gov: NCT04357938 April 22, 2020.

## Background

Hospitals have a particular responsibility towards patients as regards of protection against infections. Due to the high contagiousness of severe acute respiratory syndrome coronavirus 2 (SARS-CoV-2) [1], each infected patient, missed by reverse transcription polymerase chain reaction (RT-PCR) test before referral to general ward, carries a high risk of disease transmission and subsequent in-hospital spread with associated illness, death, and considerable follow-up costs.

To definitely exclude COVID-19 in critical areas such as hospital general ward, disease prevalence should be taken into account to set out a clear diagnostic strategy. In general, when prevalence is low (i.e. sporadic transmission patterns), a highly sensitive test is most appropriate to rule out the disease. The strategy of using a “sensitive test when the negative result rules out the disease” (SNOUT rule) is applicable [2]. Claimed a high sensitivity of chest computed tomography (CT) for COVID-19 based on earlier studies[3–5], and given a low prevalence among adults who were admitted to our university hospital emergency department for any reason but suspected of COVID-19, there would be a high probability in case of negative chest CT results that COVID-19 is absent. Therefore, low dose chest CT might usefully supplement RT-PCR tests, that, in turn, provide a high specificity [6] but might overlook SARS-CoV-2 infection in case of low viral load in the specimen [7].

The purpose of our study was to evaluate whether sensitivity of chest CT for COVID-19 in symptomatic adults admitted to the emergency department provides a sufficiently high negative predictive value to rule out COVID-19 according to the strategy of SNOUT.

## Methods

### Study design

Our prospective, single center, diagnostic accuracy cohort study was conducted in a German university hospital between March 18 and April 28, 2020. The study protocol was approved by the ethics committee of the Jena University Hospital (No. 2020-1751_1-BO). Written informed consent was obtained from all participants. The study was registered on ClinicalTrials.gov (reference number NCT04357938).

Consecutive adults (≥ 18 years) who were admitted to the emergency department for any reason and supposed to be referred to the general ward, presenting with signs and symptoms consistent with COVID-19, and those who reported on contact to individuals with SARS-CoV-19 infection within the past 14 days were eligible. Patients who were pregnant were excluded.

**Fig. 1:**
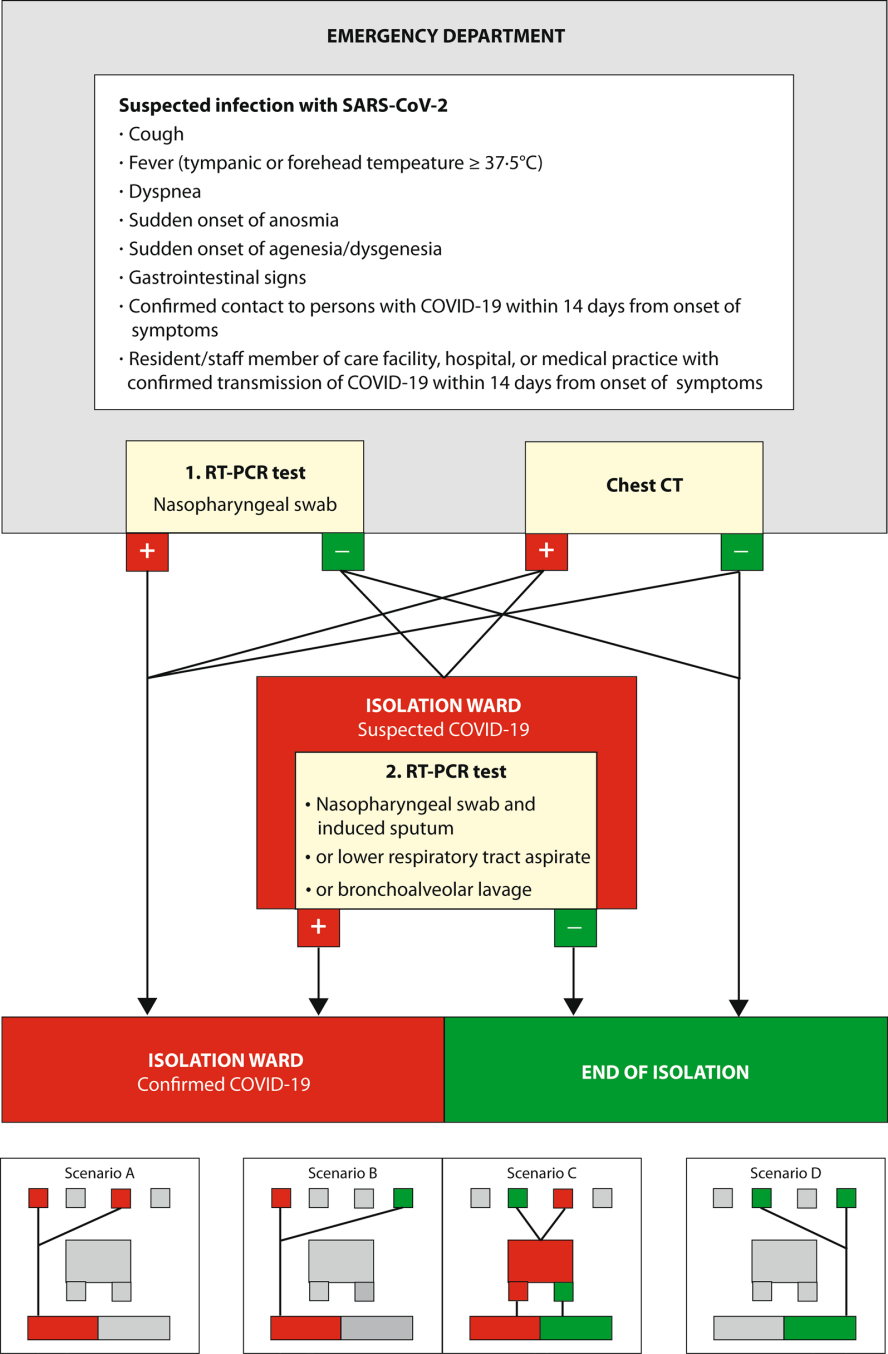
In-hospital patient flow. COVID-19, coronavirus disease 2019; CT, low-dose chest computed tomography; RT-PCR, reverse transcription polymerase chain reaction

According to hospital’s standard of care, in order to prevent spread of SARS-CoV-2 within the hospital, all adults suspected of COVID-19 underwent low-dose chest CT and specimen collection of a nasopharyngeal swab for RT-PCR at the emergency department without delay. Participants with at least one SARS-CoV-2 positive RT-PCR result or a COVID-19 positive chest CT result were referred to isolation wards. In case of a positive CT- but a negative RT-PCR result, a second RT-PCR test of nasopharyngeal swab and induced sputum, lower respiratory tract aspirate, or bronchoalveolar lavage was conducted [7]. If the second RT-PCR test was negative, participants were released from quarantine (Fig. 1). Blood samples were taken after 3 to 5 weeks from onset of symptoms to perform analysis on COVID-19 specific IgG and IgM antibodies.

### Chest CT acquisition

Computed tomography (CT) was performed on a helical multi-slice CT scanner (GE Revolution, GE Healthcare, Chicago, IL, USA). Acquisitions utilized a low dose radiation exposure protocol with a tube voltage of 120 kV and a tube current–time product that was regulated by an automated dose modulation system (SmartmA, GE Healthcare, Chicago, IL, USA) and ranged from 80 to 405 mAs. The pitch was 0.992:1 with a detector collimation width of 80 mm. The nominal reconstructed section width was 0.625 mm and the reconstruction interval 0.625 mm. Effective dose (E) was estimated from dose length product (DLP) and conversion factor k = 0.014 for adult chest exams (E = DLP*k) according to the International Commission on Radiological Protection recommendations (ICRP) [8]. Acquisitions were electronically transmitted to the central picture archiving and communication system (PACS) and independently interpreted by two senior radiologists (AM and RN) with more than 10 years of experience. Readers were blinded to clinical information and reference standard results. Structured reporting was conducted according to the radiological society of North America expert consensus statement on reporting chest CT findings related to COVID-19. The categories “typical appearance” and “nonspecific appearance” were prespecified to correspond to positive CT results and “atypical” and “negative for pneumonia” to negative CT results (Additional file 1: Table S1) [9].

### Study endpoints

The primary endpoint was sensitivity and specificity of chest CT for COVID-19. Prespecified secondary endpoints were positive and negative likelihood ratio (LR+, LR−), area under the receiver operating curve (AUROC) of chest CT, as well as posterior probability of COVID-19 after chest CT diagnosis (detailed definitions of measures of accuracy are provided in Additional file 1) [2]. Exploratory secondary endpoints were performance characteristics of specific CT patterns and distributions of abnormal findings in participants’ lungs. Evaluation of diagnostic accuracy was based on either the reference standard of RT-PCR test only or the reference standard of RT-PCR and AB test were available (either of which may have identified SARS-CoV-2 infection), (methods of assessment of reference standard are provided in Additional file 1). We included AB test results into the reference standard to increase sensitivity. Indeterminate AB test results or missing data together with positive RT-PCR results constituted positive combined reference standard results, and, together with negative RT-PCR test results, constituted negative combined reference standard results.

### Statistical analysis

Based on daily situation reports on coronavirus incidence in each federal state of Germany, published by the Robert Koch Institute, Berlin, Germany, and taking into account a tenfold increase due to unreported cases, we assumed a COVID-19 prevalence of 1% in the Thuringian population, and of 10% in symptomatic patients admitted to our emergency department. The number of participants required to achieve a precision of a two-sided 95% confidence interval (CI) including margins of error of at most 10% on either side for an assumed sensitivity of 90% along with a specificity of 80%, based on previous findings [3, 4], was calculated with 346 [10]. Due to urgent need to confirm capability of chest CT to reliably rule out COVID-19 to prevent in-hospital spread, preliminary analysis was conducted on achievement of 50% of the intended sample size. Predictive values were calculated based on the theorem of Bayes using Fagan’s nomogram [11]. Clopper-Pearson CIs were provided for sensitivity and specificity, CIs calculated using the Log method for likelihood ratios, and logit CIs for predictive values. Additionally, receiver operating characteristic analysis was conducted to determine AUROCs. A two-sided *P*-value of < 0.05 was considered statistically significant. Analysis was performed using XLSTAT (Version 2015.6.01.24026, Addinsoft, Paris, France).

## Results

### Study participants

**Fig. 2:**
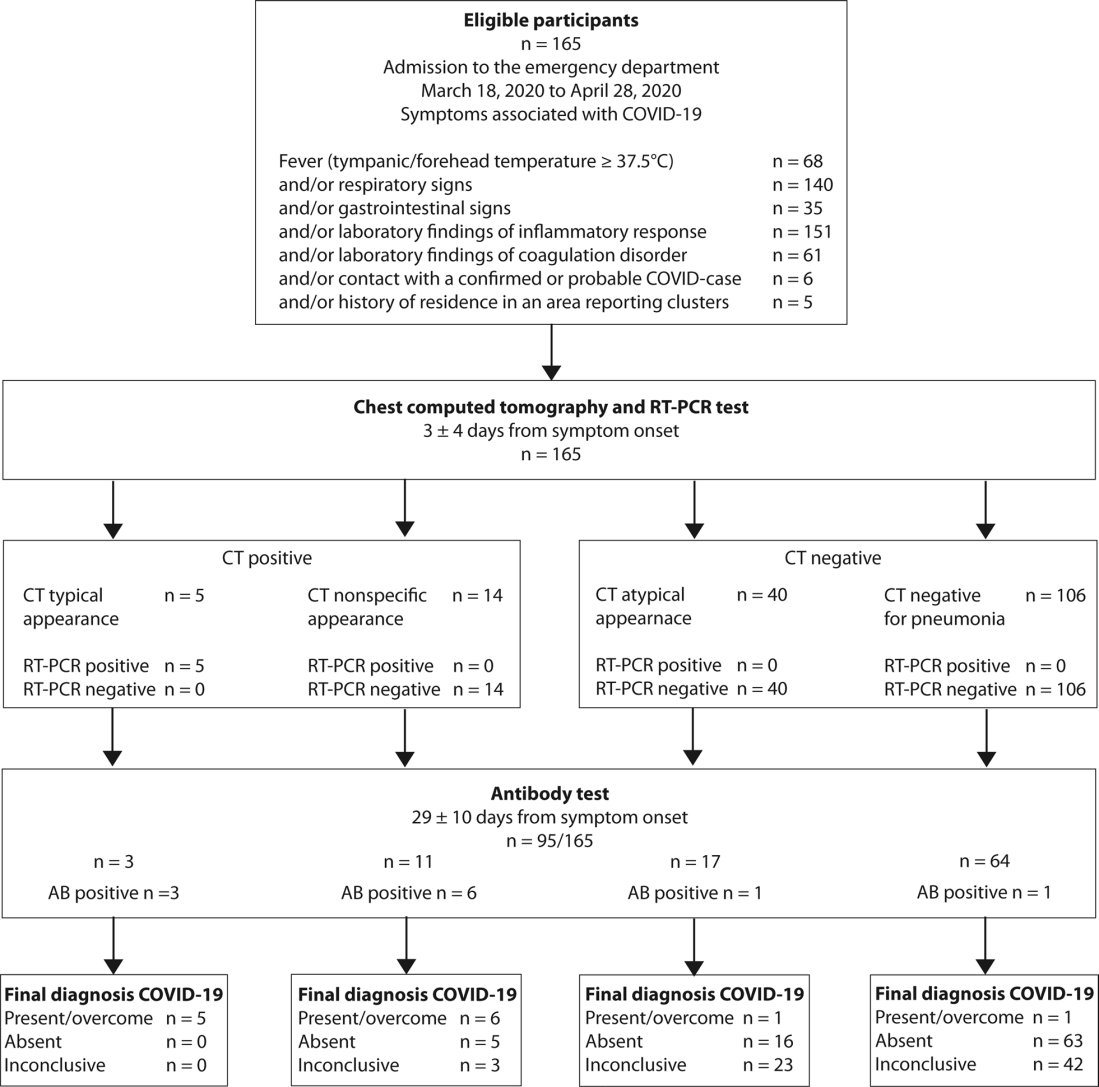
Flow of participants through the study. AB, antibody test; COVID-19, coronavirus disease 2019; CT, low-dose chest computed tomography; RT-PCR, reverse transcription polymerase chain reaction

A total of 165 patients who were supposed to be referred from the emergency department to the general ward and suspected to be infected with SARS-CoV-2 were included in the study (Table 1). Symptom onset was 3 ± 4 days before admission (Table 2). All participants underwent chest CT and at least one RT-PCR-test. Specimen for the first RT-PCR test were nasopharyngeal swab and induced sputum in 47.3% (78 of 165), nasopharyngeal swab in 36.4% (60 of 165), lower respiratory tract aspirate in 12.7% (21 of 165), pharyngeal swab only in 2.4% (four of 165) and sputum only in 1.2% (two of 165) of participants. Samples for the second RT-PCR test were available in 78.6% (11 of 14) of participants with positive chest CT result along with negative first RT-PCR test result (Additional file 1: Table S2, Fig. S1). Antibody test was conducted at 29 ± 10 days after symptom onset in 57.6% (95 of 165) of participants. Prevalence of COVID-19 was 7.9% (13 of 165 patients), (Fig. 2). A comparison of demographics, comorbidities, symptoms, and laboratory findings of participants with confirmed COVID-19 with those without COVID-19 is shown in the Tables 1, 2, 3. Except for an increased frequency of preceding contact to persons with COVID-19, a lower heart rate, and an increased level of fibrinogen in COVID-19 positive participants, no differences between groups could be demonstrated. Proportion of participants with fever, cough, fatigue, and/or dyspnea did not differ significantly between COVID-19 positive and negative participants. Findings from clinical evaluation, chest CT-, and laboratory diagnostics of individual participants who were diagnosed as COVID-19 positive by means of any test and/or chest CT are shown in Additional file 1: Tables S3 and S4.

**Table 1:** Participant demographics and comorbidities

Characteristics	All participants (N = 165)	COVID-19 present/overcome (n = 13)	COVID-19 absent (n = 152)	*P* value
Age, years	71.0 ± 15.8	72.1 ± 11.5	70.9 ± 16.2	.97
Sex				
Male	93 (56.4)	10 (76.9)	83 (54.6)	.12
Female	72 (43.6)	3 (23.1)	69 (45.4)	.12
History of possible exposure to Sars-CoV-2	11 (16.9)	4 (30.8)	7 (4.6)	.006
Smoker (former or current)	41/142 (28.9)	2/10 (20.0)	39/132 (29.5)	.72
Body mass index, kg/m^2^	27.8 ± 8.1	28.9 ± 4.5	27.7 ± 8.5	.26
Chronic pulmonary disease	38 (23.0)	5 (38.5)	33 (21.7)	.18
Ischemic heart disease	48 (29.1)	5 (38.5)	43 (28.9)	.53
Heart failure	85 (51.5)	7 (53.8)	78 (51.3)	> .99
Cerebrovascular disease	47 (28.5)	1 (7.7)	46 (30.3)	.11
Diabetes	52/164 (31.7)	4/12 (33.3)	51 (33.6)	> .99
Currently uncontrolled	22/164 (13.4)	2/12 (16.7)	20 (13.2)	.67
Well adjusted	33/164 (20.1)	2/12 (16.7)	31 (20.4)	
Malignancy	45 (27.3)	5 (38.5)	39 (25.7)	.34
Current	26 (15.8)	3 (23.1)	23 (15.1)	.68
Previous	19 (11.5)	3 (23.1)	16 (10.5)	.68
Arterial hypertension	131 (79.4)	11 (84.6)	120 (78.9)	> .99
Chronic renal failure	52 (31.5)	5 (38.5)	47 (30.9)	.11
Immune-compromised^a^	71 (43.0)	5 (38.5)	66 (43.4)	.78
Chronic liver disease	23 (13.9)	2 (15.4)	21 (13.8)	> .99

Data are mean ± SD or n (%)

^a^Participants under immunosuppressive therapy and those with tumors, type 1 diabetes, advanced liver cirrhosis, or transplant were considered immune-compromised

**Table 2:** Participant symptoms

Symptoms	All participants (N = 165)	COVID-19 present/overcome (n = 13)	COVID-19 absent (n = 152)	*P* value
Time from symptom onset, days	3 ± 4	5 ± 5	3 ± 4	.16
Body temperature (tympanic, forehead), °C	37.4 ± 1.0	37.1 ± 1.3	37.4 ± 1.0	.24
≥ 37.5 °C	68/163 (41.7)	4 (33.3)	64/149 (43.0)	.47
O_2_—saturation ≤ 92%	36/162 (22.2)	5/12 (41.7)	31/150 (20.7)	.14
Cough	78 (47.3)	6 (46.2)	72 (47.4)	> .99
Dyspnea	81 (49.1)	6 (46.2)	75 (49.3)	> .99
Breathing rate ≥ 24/min	21/76 (27.6)	1/6 (16.7)	20/70 (28.6)	> .99
Breathing noise, pathological findings	62 (37.6)	6 (46.2)	56 (36.8)	.56
Attenuated	19 (11.5)	1 (7.7)	18 (11.8)	.44
Ambient noise	43 (26.1)	5 (38.5)	38 (25.0)	
Percussion, pathological findings	29 (17.6)	1 (7.7)	28 (18.4)	.47
Attenuated	17 (10.3)	0 (0.0)	17 (11.2)	.23
Tympanic resonance	12 (7.3)	1 (7.7)	11 (7.2)	
Sputum	45 (27.3)	2 (15.4)	43 (28.3)	.52
Glassy white	11 (6.7)	0 (0.0)	11 (7.2)	.70
Foaming	15 (9.1)	1 (7.7)	14 (9.2)	
Purulent	19 (11.5)	1 (7.7)	18 (11.8)	
Rhinorrhea	12 (7.3)	1 (7.7)	11 (7.2)	> .99
Sore throat	17 (10.3)	1 (7.7)	16 (10.5)	> .99
Fatigue	46 (27.9)	6 (46.2)	40 (26.3)	.19

Data are mean ± SD or n (%)

**Table 3:** Laboratory findings

Findings	All participants (N = 165)	COVID-19 present/overcome (n = 13)	COVID-19 absent (n = 152)	*P* value
Leucocyte count, × 10^9^ per L	11.5 ± 9.4	12.9 ± 9.5	11.4 ± 9.4	.77
< 10	82 (49.7)	6 (46.2)	76 (50.0)	.79
≥ 10	83 (50.3)	7 (53.8)	76 (50.0)	
Lymphocyte count, × 10^9^ per L, (n = 90)	1.2 ± 0.7	1.5 ± 0.9	1.2 ± 0.6	.31
< 1.0	41/90 (45.6)	4/9 (44.4)	37/81 (45.7)	.94
≥ 1.0	49/90 (54.4)	5/9 (55.6)	44/81 (54.3)	
C-reactive protein, mg/L, (n = 164)	68.9 ± 74.0	95.1 ± 88.2	66.6 ± 72.6	.34
Procalcitonin, ng/mL, (n = 93)	1.6 ± 4.6	0.6 ± 0.8	1.7 ± 4.8	.99
≥ 0.5	29/93 (31.2)	3/7 (42.9)	26/86 (30.2)	.67
IL-6, pg/mL, (n = 33)	154.7 ± 255.2	29.4 ± 20.8	177.9 ± 272.9	.34
d-dimers, mg/L, (n = 63)	1.0 ± 1.4	0.5 ± 3.6	1.1 ± 1.5	.74
> 0.5	29/60 (48.3)	2/5 (40.0)	27/55 (40.1)	> .99
Fibrinogen, g/L, (n = 72)	4.2 ± 1.7	4.6 ± 1.5	4.2 ± 1.7	.26
> 5.0	22/72 (30.6)	5/8 (62.5)	17/64 (26.6)	.051
Hemoglobin, g/L	119.7 ± 244.0	125. 1 ± 244.0	119.2 ± 249.0	.38
High-sensitive cardiac troponin I, pg/mL				
Male (n = 53)	79.8 ± 221.0	11.0 ± 7.6	98.7 ± 196.8	.78
Female (n = 39)	90.5 ± 188.9	42.9 ± 51.1	81.8 ± 226.8	.25
> 34 (male), > 16 (female)	35/92 (38.0)	1/7 (14.3)	34/85 (40.0)	.25
NT-proBNP, ≥ 500 pg/mL, (n = 55)	18/55 (34.6)	2/3 (66.7)	16/52 (30.8)	.25
Serum Ferritin, μg/L, (n = 57)	768.0 ± 1253.9	705.4 ± 581.4	775.3 ± 1314.0	.25
> 300	26/56 (46.4)	4/6 (66.7)	22/50 (44.0)	.29
Creatinine, μmol/L, (n = 160)	122.3 ± 90.7	116.3 ± 47.9	122.8 ± 93.7	.40
> 150 (male), > 124 (female)	32/159 (20.1)	3/13 (23.1)	29/146 (19.9)	.73

Data are mean ± SD or n (%)

IL-6, interleukin-6

### Diagnostic accuracy of chest CT

**Fig. 3:**
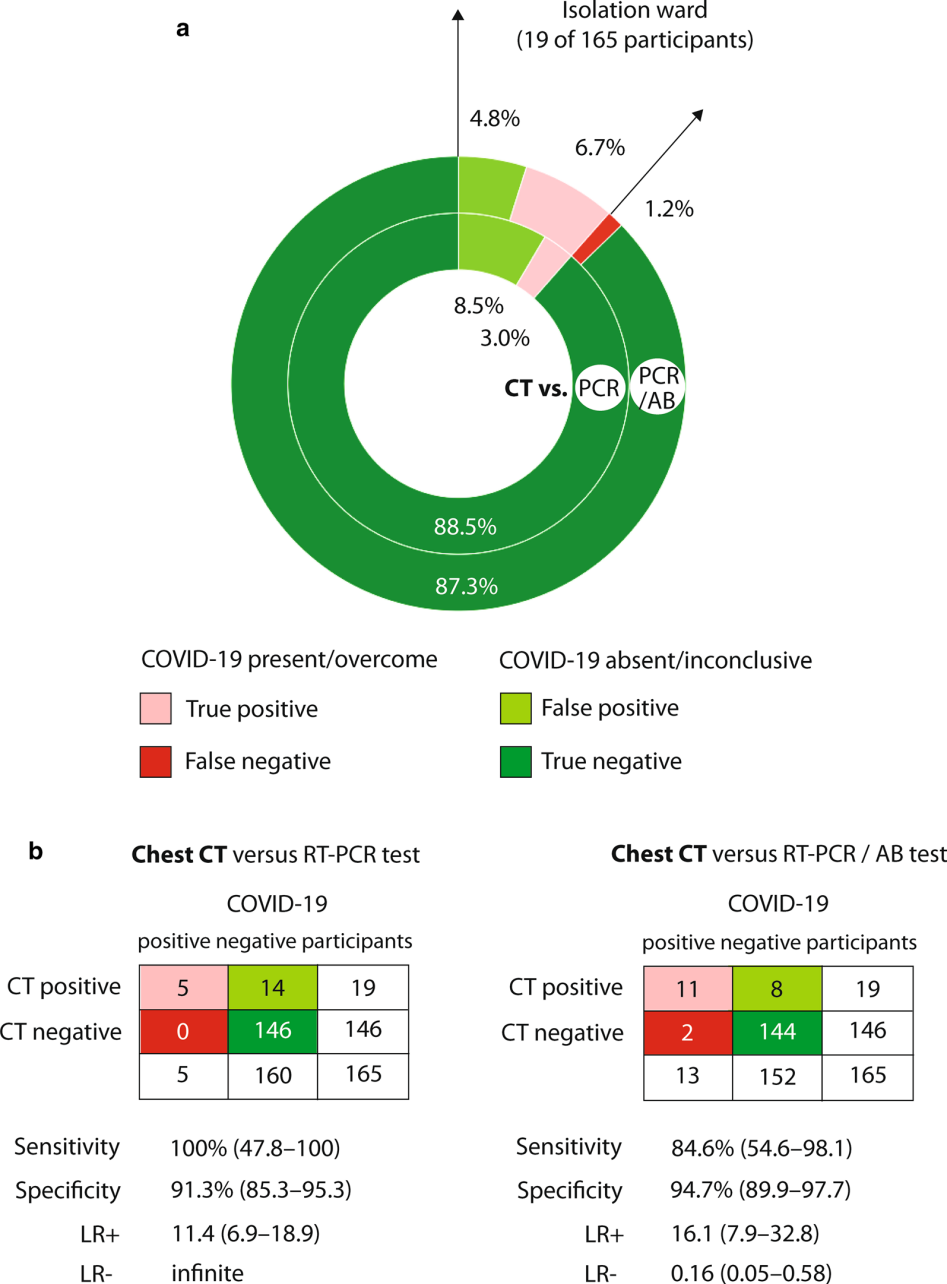
Diagnostic characteristics of low-dose chest CT and impact on participant admission to isolation ward. **a** Doughnut chart represents proportions and **b** contingency tables frequencies of true positive, false negative, true negative, and false negative diagnosed participants. Chest CT diagnostic performance is given for both the reference standard of RT-PCR test only and the reference standard of RT-PCR and/or AB test. Test characteristics are presented with 95% confidence intervals. AB, antibody test; COVID-19, coronavirus disease 2019; CT, low-dose chest computed tomography; LR + , positive likelihood ratio; LR-, negative likelihood ratio; RT-PCR, reverse transcription polymerase chain reaction

Sensitivity of chest CT for COVID-19 was 100% (95% confidence interval [CI], 47.8–100) using both RT-PCR and AB test results as reference standard, and 84.6% (95% CI, 54.6–98.1) using both RT-PCR and/or AB test results as reference standard. Specificity was 91.3% (95% CI, 85.3–95.3) and 94.7% (95% CI, 89.9–97.7), respectively. Chest CT accurately identified either five (3.0%) or 11 (6.7%) of 165 participants with COVID-19 depending on whether RT-PCR test only or both RT-PCR- and AB test were applied as standard reference (Fig. 3).

Six participants with positive chest CT result showed a negative RT-PCR- but a positive AB test result. In 2 RT-PCR/AB positive participants without respiratory symptoms, chest CT identified COVID-19 pneumonia. Two participants (1.2%) with positive AB test result showed no pathology in chest CT. One of these participants was a 60-year-old male presented with respiratory symptoms that started 21 days ago. His chest CT reveals pulmonary emphysema and pericardial effusion. Blood sample for the AB test was collected five weeks after onset of symptoms. The second “false negative” participant was an 83-year-old female, presented with fever and weakness that started at the day of admission. Chest CT revealed fibrous stripes. AB test was conducted 15 days from onset of symptoms (Additional file 1: Table S3).

**Fig. 4:**
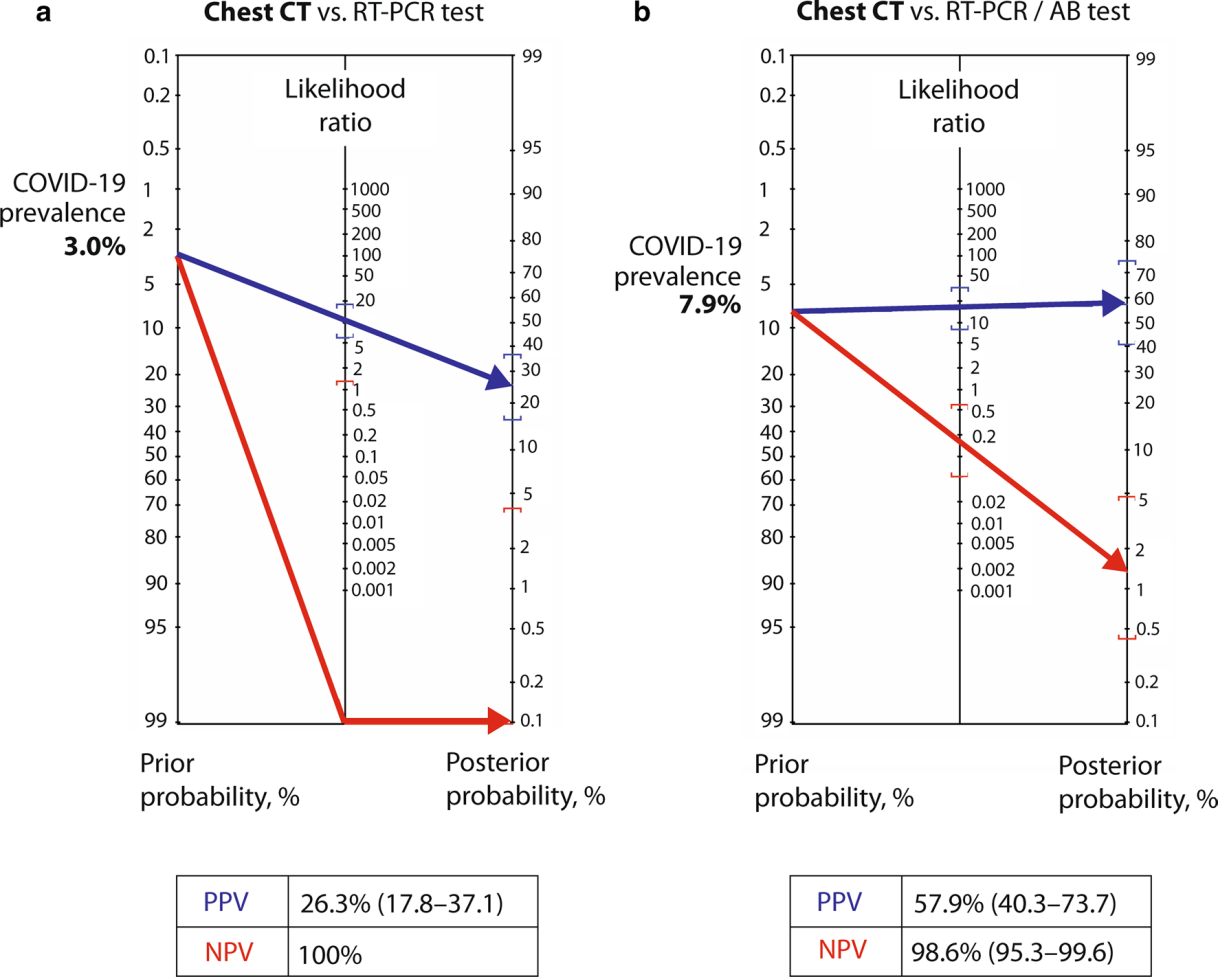
Fagan`s nomogram showing probability of COVID-19 infection after chest CT diagnosis. **a** Probabilities were calculated based on the reference standard of RT-PCR test only or **b** RT-PCR and/or AB test. Positive CT diagnosis (blue arrow) refers to typical or nonspecific appearance, and negative CT diagnosis (red arrow) to atypical or negative appearance of CT scan. Precision is given as 95% confidence interval. Disease prevalence is derived from the number of COVID-19 positive and negative participants. AB, antibody test; CT, low-dose chest computed tomography; LR+, positive likelihood ratio; LR−, negative likelihood ratio; NPV, negative predictive value; PPV, positive predictive value; RT-PCR, reverse transcription polymerase chain reaction

Using the standard reference of RT-PCR only, the low pre-test probability (prevalence 3.0%) together with a positive likelihood ratio of 11.4 (95% CI 6.9–18.9) led to a positive predictive value of 26.3% (95% CI 17.8–37.1) and, with an infinite negative likelihood ratio, to a negative predictive value of 100% (Fig. 4a). Considering both RT PCR- and AB test results, pre-test probability (prevalence 7.9%) and likelihood ratios increased (LR+: 16.1 [95% CI 7.9–32.8], LR-: 0.16 [95% CI 0.05–0.58], respectively) and thus, finally, chest CT achieved a positive predictive value of 57.9% (95% CI 40.3–73.7) and a negative predictive value of 98.6% (95% CI 95.3–99.6), (Fig. 4b).

Chest CT had an AUROC of 0.90 (95% CI 0.78–1.0), *P* < 0.001 regarding the dichotomous outcome of COVID-19-positive or -negative result, and of 0.92 (95% CI 0.82–1.0), *P* < 0.001 regarding the categorical outcome of the four-stage COVID-19 chest CT imaging classification (Additional file 1: Fig. S2). The ROC curve confirmed the RSNA classification category of “nonspecific appearance” as optimal cut-off value. Chest CT in addition to RT-PCR tests increased the AUROC compared to RT-PCR test alone by 0.24 (95% CI 0.1–0.4), *P* = 0.002 (Additional file 1: Fig. S3). Additionally, AUROCs of specific CT patterns and distributions of abnormal findings confirmed general discriminating power of chest CT for COVID-19 (ground-glass-opacity: 0.79 [SE 0.06, *P* < 0.001]; fibrous stripes (interlobular septal thickening): 0.67 [SE 0.07, *P* = 0.02]; unspecific patterns: = 0.65 [SE 0.07, *P* = 0.04]; bilateral distribution: 0.68 [SE 0.68, *P* = 0.001]; multi-lobar distribution (upper and lower lobes): 0.67 [SE 0.67, *P* = 0.01]) (Additional file 1: Figs. S4 and S5**).**

The computed tomography dose index (CDTI) was 5.0 ± 3.2 mGy and the irradiated length 31.7 ± 3.1 cm. From this we deduced a dose length product (DLP) of 188.3 ± 107.9 mGy*cm and an estimated effective dose (E) of 2.6 ± 1.5 mSv per chest CT investigation.

## Discussion

In our study, we evaluated diagnostic accuracy of low-dose chest CT in adults suspected of COVID-19 to be referred from the emergency department to general wards. Due to the low prevalence of COVID-19 in our study population, negative chest CT allowed to rule out COVID-19 with high certainty. Both typical and nonspecific CT features of COVID-19 identified participants with SARS-CoV-2 infection that would have been missed by RT-PCR test alone.

When COVID-19 prevalence is low, and thus, diagnostic strategy of SNOUT should be adopted, even the RT-PCR test may achieve an acceptable negative predictive value to exclude COVID-19. Although, its sensitivity is lower compared to chest CT, particularly in elderly patients [6, 12]. However, costs of false negative results associated with SARS-CoV-2 transmission are high. Concomitant low dose chest CT in adults admitted to emergency departments for a variety of reasons who have symptoms and/or previous contact to SARS-CoV-2 infected individuals may cover the lack of sensitivity and provide a more reliable negative predictive value along with reasonable radiation exposure. However, an earlier study showed that sensitivity of chest CT decreased in case of broad distribution of disease severity and high proportion of comorbidities [12].

As reported earlier, chest CT is able to identify COVID-19 in participants without respiratory symptoms [13, 14]. In our study, chest CT identified two participants without respiratory symptoms as COVID-19 positive. The first participant had no symptoms but had been referred from another hospital and thus, was suspected of being affected. COVID-19 diagnosis was confirmed by RT-PCR test. The second participant suffered from fever, weakness, headache, and dizziness for one day. RT-PCR test was negative. Later, COVID-19 was confirmed by AB test. However, we cannot be quite sure at which time the patient had been contagious.

Previous studies found false negative CT results particularly during the first four days after symptom onset [15, 16] and neither RT-PCR test nor chest CT can detect incubating SARS-CoV-2 infection. Two participants of our study who were diagnosed COVID-19 negative by chest-CT had positive AB test results and thus were classified as “false negatives”. In one of these participants, respiratory symptoms started 3 weeks before chest CT, and AB test was conducted 5 weeks after symptom onset. In this participant, pulmonary emphysema might have superimposed typical COVID-19 patterns on CT. Even an infection after chest CT cannot be excluded. In the second false negative participant, AB test was conducted 15 days after onset of fever and weakness. As serum conversion occurs at the earliest 7 days, but more reliably 14 days from symptom onset [17], in this participant, COVID-19 might have been overcome or present without pneumonia at the day of symptom onset and chest CT. A recent meta-analysis reported on 8.4% of normal CT findings in COVID-19 patients [18]. The SNOUT rule is not applicable where prevalence is high. A previous study in Chinese regions with COVID-19 prevalence of 85% by RT-PCR found a chest CT sensitivity of 93% resulting in a NPV of only 42% [19].

A total of 14 CT-positive participants in our study were released from isolation after negative second RT-PCR test result. However, six of them later turned out to be AB-positive. This might have been due to overcome disease at the time of admission or to lack of sampling from deeper airways. In our study we decided to complement results from the RT-PCR test by results from AB-test to improve the sensitivity of the composite reference standard. Thereby, we accepted the remaining uncertainty regarding the exact time of COVID-19 infection in favor of an increased probability to identify the highest possible number of contagious patients. In retrospect, as a precaution, we would recommend, to keep all CT-positive patients in quarantine until tested negative with a second PCR-test from bronchoalveolar specimen.

Specificity of chest CT was considerably lower than known from RT-PCR tests. Thus, chest CT does not appear appropriate for screening or as first line diagnostic test to “rule in” COVID-19. Previous studies reported on specificity that ranges from 25% to 100% [3–5, 19]. However, typical CT imaging features including bilateral and multi-lobar distribution of ground-glass opacity with or without crazy paving pattern and fibrous strips were consistent with previous reports and thus may be considered as characteristic of COVID-19 [18, 20, 21]. However, at a low prevalence, positive predictive value of chest CT is only moderate. Broad distribution of COVID-19 severity and high frequency of different severe alternative diagnosis in the clinical setting of the university hospital emergency department may also have contributed to an increased frequency of false positives. Moreover, in particular in flue seasons with high influenza prevalence there probably will be more false positive chest CT results. Thus, performance of radiologists [5] to distinguish COVID-19 from other viral infections will be crucial.

Our study has limitations. First, due to social lockdown, instituted by the German government and the accompanied considerably reduced COVID-19 incidence, we decided to prematurely terminate recruitment. Therefore, number of participants did not achieve the targeted sample size and precision of endpoints remained low. In addition, only around half of the participants who underwent chest CT were assessed by AB test. In these participants, the RT-PCR result alone applied as reference standard. Furthermore, both reference standards have limitations. Sensitivity of RT-PCR depends on the viral load that differs according to the time of sampling, to the specimen, specimen handling, and even among individual participants [7]. Detection of specific antibodies is insufficient to permit a conclusion on the exact time of infection. In participants with positive AB test result, it is therefore uncertain whether a confirmed SARS-CoV-2 infection was present or already overcome at the time of chest CT. Finally, we did not collect data on dysgeusia and anosmia because these symptoms were not known to be typical for COVID-19 at the time of inclusion.

## Conclusion

Results of our study suggest that low dose chest CT is well suited to rule out COVID-19 where prevalence is low (strategy of SNOUT), and thus, may supplement RT-PCR testing in emergency departments to guide the choice of referral to general wards. From our study, we derived to precautionary isolate chest CT positive patients in single patient rooms—even in case of negative RT-PCR results or confirmed alternative diagnosis.

## Supplementary Information

**Additional file 1:** Additional tables and figures.

## Data Availability

The datasets generated during and/or analyzed during the current study are available from the corresponding author on reasonable request.

## Abbreviations

AB test: Antibody test
AUROC: Area under the receiver operating curve
COVID-19: Corona virus disease 2019
RT-PCR: Reverse transcription polymerase chain reaction
SARS-CoV-2: Severe acute respiratory syndrome coronavirus 2
SNOUT: Sensitive test when the negative result rules out the disease
