# Supplementary material for: Ruling out COVID-19 by chest CT at emergency admission when prevalence is low: the prospective, observational SCOUT study
Source: Respir Res. 2021 Jan 12;22:13. doi: 10.1186/s12931-020-01611-w (PMC7802980; doi:10.1186/s12931-020-01611-w)
Supplement: Supplementary file 1 — Additional file 1: Additional tables and figures. [file 12931_2020_1611_MOESM1_ESM.docx]

**Additional file**

**Ruling out COVID-19 by chest CT at emergency admission when prevalence is low:**

**the prospective, observational SCOUT study**

Ulf Teichgräber^1^, Amer Malouhi^1^, Maja Ingwersen^1^, Rotraud Neumann^1^, Marina Reljic^2^, Stefanie Deinhardt-Emmer^3^, Bettina Löffler^3^, Wilhelm Behringer^4^, Jan-Christoph Lewejohann^4^, Andreas Stallmach^2^, Philipp Reuken^2^

^1^Departments of Radiology, Friedrich-Schiller-University, Jena University Hospital, Am Klinikum 1, 07747 Jena, Germany

^2^Department of Internal Medicine, Friedrich-Schiller-University, Jena University Hospital, Am Klinikum 1, 07747 Jena, Germany.

^3^Institute of Medical Microbiology, Friedrich-Schiller-University, Jena University Hospital, Am Klinikum 1, 07747 Jena, Germany.

^4^Department of Emergency, Friedrich-Schiller-University, Jena University Hospital, Am Klinikum 1, 07747 Jena, Germany.

**Methods**

**Measures of accuracy**

Sensitivity represented the proportion of participants with COVID-19 who were correctly identified by chest CT (true positive rate), and specificity the proportion of participants without COVID-19 who were correctly identified by chest CT (true negative rate). Positive likelihood ratio (LR+) was defined as sensitivity/(1-specifity) and negative likelihood ratio (LR-) as (1-sensitivity)/specificity. LR+ denotes how much more likely a positive diagnosis was among COVID-19 participants than it was among those without COVID-19. Reversely, LR- denotes how much more likely a negative diagnosis was among participants in whom COVID-19 was absent than it was among COVID-19 participants. Posterior probability of present/overcome COVID-19 in case of positive chest CT result (positive predictive value [PPV]) and, reversely, absent COVID-19 in case of a negative chest CT diagnosis (negative predictive value [NPV]) were derived from likelihood ratios and prevalence (pre-test probability) [1].

**Reference standard**

For identification of SARS-CoV-2 from respiratory specimen, we extracted nucleic acids by using an automated EZ1-system (Qiagen, Hilden, Germany). The isolated and purified RNA was used for RT-PCR to determine the E-gene. For this purpose, TIB MOLBIOL RT-PCR (Berlin, Germany) was applied on a LightCycler 480 Roche system (Roche Diagnostics, Mannheim, Germany) with a specificity of 100% and a sensitivity of 5.2 copies/reaction (95% CI, 3.7 -9.6) [2].

Specific IgG and IgM antibodies were detected in participant’s serum by applying an enzyme-linked immunosorbent assay. We used the EDI™ Novel Coronavirus COVID-19 ELISA Kit (Epitope diagnostics Inc., San Diego, CA, USA) with a sensitivity of 100% and a specificity of 88% [3].

Assessors of RT-PCR and antibody (AB) tests were blinded to clinical information and chest CT results.

| **Table S1** COVID-19 pneumonia imaging classification | | |
| --- | --- | --- |
| COVID-19 pneumonia imaging classification | CT Findings | Rationale |
| Typical appearance | - Peripheral, bilateral, GGO with or without consolidation or visible intralobular lines (“crazy-paving”) - Multifocal GGO of rounded morphology with or without consolidation or visible intralobular lines (“crazy-paving”) - Reverse halo sign or other findings of organizing pneumonia (seen later in the disease) | Commonly reported imaging features of greater specificity for COVID-19 pneumonia. |
| Nonspecific appearance | Absence of typical features  AND presence of:   - Multifocal, diffuse, perihilar, or unilateral GGO with or without consolidation lacking a specific distribution and are non-rounded or non-peripheral. - Few very small GGO with a non-rounded and non-peripheral distribution | Nonspecific imaging features of COVID-19 pneumonia. |
| Atypical appearance | Absence of typical or nonspecific features AND presence of:   - Isolated lobar or segmental consolidation without GGO - Discrete small nodules (centrilobular, “tree- in-bud”) - Lung cavitation - Smooth interlobular septal thickening with pleural effusion | Uncommonly or not reported features of COVID-19 pneumonia. |
| Negative for pneumonia | No CT features to suggest pneumonia. | No features of pneumonia |
| COVID-19, corona virus disease 2019; GGO, ground glass opacity.  Adapted from Simpson 2020 [4] | | |

| **Table S2.** Location and Time of Specimen Collection in Participants with Negative RT-PCR but Positive AB and/or Positive CT | | | | | |
| --- | --- | --- | --- | --- | --- |
| **RT-PCR negative and**  **CT and/or AB positive participants** | | **1. RT-PCR** | | **2. RT-PCR** | |
|  |  | Days from symptom onset | Specimen | Days from symptom onset | Specimen |
| CT positive  AB positive | Male, 70 years | 1 day | Nasopharyngeal | NA | NA |
|  | Male, 81 years | 1 day | Nasopharyngeal  Induced sputum | 2 days | Bronchoalveolar |
|  | Male, 82 years | 14 days | Nasopharyngeal | 19 days | Nasopharyngeal |
|  | Male, 86 years | 20 days | Nasopharyngeal | 21 days | Nasopharyngeal |
|  | Male, 85 years | 7 days | Nasopharyngeal | NA | NA |
|  | Male 48 years | 7 days | Nasopharyngeal  Induced sputum | 8 days | Nasopharyngeal |
| CT negative  AB positive | Male 60 years | 21 days | Nasopharyngeal | NA | NA |
|  | Female 83 years | Same day | Nasopharyngeal | NA | NA |
| CT positive  AB negative | Male 58 years | 6 days | Nasopharyngeal | NA | Nasopharyngeal |
|  | Female 86 years | 2 days | Nasopharyngeal | 4 days | Nasopharyngeal  Induced sputum |
|  | Male 70 years | 3 days | Nasopharyngeal  Induced sputum | NA | NA |
|  | Female 76 years | 1 day | Lower respiratory tract aspirate | 2 days | Lower respiratory tract aspirate |
|  | Male 72 years | 2-3 days | Lower respiratory tract aspirate | 3-4 days | Lower respiratory tract aspirate |
|  | Male 60 years | 5 days | Lower respiratory tract aspirate | 6 days | Lower respiratory tract aspirate |
|  | Female 91 years | 10 days | Lower respiratory tract aspirate | 11 days | Lower respiratory tract aspirate |
|  | Female 86 years | 12 days | Nasopharyngeal  Induced sputum | 13 days | Nasopharyngeal |
| AB, antibody test; NA, not available; CT, computed tomography; RT-PCR, reverse transcription polymerase chain reaction test | | | | | |

| **Table S3.** Characteristics of participants diagnosed COVID-19 positive by means of chest CT and/or RT-PCR and/or AB-test | | | | | | | | | |
| --- | --- | --- | --- | --- | --- | --- | --- | --- | --- |
| **Positive diagnostic test** | **Participant** | **Chest CT findings** | **Symptom onset before admission** | **Contact and/or association to cluster** | **Fever ^a^**  **(≥ 37.5 C°)** | **Respiratory symptoms** | **Gastro-**  **intestinal symptoms** | **General**  **symptoms** | **Oxygen satu-ration** |
| CT, RT-PCR, AB | Male, 61 years  Congestive heart failure | GGO, CP, UP,  peri-bronchial | 3 days | Yes | No | Dyspnea, ambient breathing noise, foaming sputum, cough | No | Weakness, headache | 98% |
|  | Female, 76 years | GGO, CP, UP, FS, SL,  peripheral and central | A few weeks | 14 days before admission | No | Cough | No | Slightly reduced general condition | 96% |
|  | Male, 76 years  Congestive heart failure | GGO, UP, FS, peripheral and central | 10 days | Yes | No | Dyspnea, cough | No | No | 92% |
| CT, RT-PCR | Female, 65 years, AB NA | GGO, peripheral and central | 3 days | Yes | Yes | Dyspnea, ambient breathing noise, purulent sputum, cough | Diarrhea | Weakness, dizziness | 88% |
|  | Male, 69 years, AB NA | GGO, UP, FS, SL, peripheral | No symptoms | Referral from another hospital | No | No | No | No | 96% |
| CT, AB | Male, 70 years | FS, peripheral and central | 1 day | No | Yes | No | No | Weakness, headache, dizziness | 96% |
|  | Male, 81 years  Congestive heart failure | GGO, UP, FS, SL, peripheral and central | 1 day | No | No | Attenuated breathing noise, cough | No | Weakness, headache, dizziness | 92% |
| ***(Continues)*** | | | | | | | | | |
| **Table S3 *(Continued)*** Characteristics of participants diagnosed COVID-19 positive by means of chest CT and/or RT-PCR and/or AB-test | | | | | | | | | |
| **Positive diagnostic test** | **Participant** | **Chest CT findings** | **Symptom onset before admission** | **Contact and/or association to cluster** | **Fever ^a^**  **(≥ 37.5 C°)** | **Respiratory symptoms** | **Gastro-**  **intestinal symptoms** | **General**  **symptoms** | **Oxygen satu-ration** |
|  | Male, 82 years  Congestive heart failure | GGO, UP, FS,  peripheral and central | 14 days | No | No | Dyspnea, ambient breathing noise | No | Weakness, arthralgia | 91% |
|  | Male, 86 years  Congestive heart failure | GGO, UP, N, peripheral | 20 days | No | Yes | Ambient breathing noise, rhinorrhea, | No | Weakness, dizziness | 97% |
|  | Male, 85 years | GGO, TB, UP, peripheral and central | 7 days | No | No | Dyspnea, ambient breathing noise, tympanic resonance | No | Weakness, dizziness, arthralgia | 84% |
|  | Male, 48 years | GGO, TB, peripheral | 7 days | No | No | Sore throat | Diarrhea | No | 98% |
| AB | Male, 60 years | Pulmonary emphysema, pericardial effusion | 21 days | No | No | Dyspnea, cough | No | Weakness, dizziness | NA |
|  | Female, 83 years | FS, peripheral and central | Same day | No | Yes | No | No | Weakness | 100% |
| CT  (false positive) | Male, 58 years,  AB NA | GGO, CP, UP, SL, peripheral and central | 6 days | No | No | Sore throat, cough | No | Weakness | 97% |
|  | Female, 86 years,  AB NA  Congestive heart failure, exacerbating COPD | GGO, CP, UP, N, peripheral | 2 days | No | Yes | Dyspnea, cough, ambient breathing noise, tympanic resonance, purulent sputum | No | Weakness, dizziness | 97% |
| ***(Continues)*** | | | | | | | | | |
| **Table S3 *(Continued)*** Characteristics of participants diagnosed COVID-19 positive by means of chest CT and/or RT-PCR and/or AB-test | | | | | | | | | |
| **Positive diagnostic test** | **Participant** | **Chest CT findings** | **Symptom onset before admission** | **Contact and/or association to cluster** | **Fever ^a^**  **(≥ 37.5 C°)** | **Respiratory symptoms** | **Gastro**  **-intestinal symptoms** | **General**  **symptoms** | **Oxygen satu-ration** |
|  | Male, 70 years,  AB neg  Congestive heart failure | GGO, peripheral and central | 3 days | No | Yes | No | No | Weakness, dizziness | 95% |
|  | Female, 76 years,  AB neg  Congestive heart failure | GGO, UP, peripheral and central | 1 day | No | No | Dyspnea, cough, sore throat | NA | Weakness, dizziness | 92% |
|  | Male, 72 years,  AB neg | GGO, UP, peripheral and central | 2-3 days | No | Yes | No | Vomiting | Weakness, dizziness | 95% |
|  | Male, 60 years,  AB neg  Metastatic colorectal  cancer, pericarditis | GGO, UP, N, peripheral and central | 5 days | No | No | Dyspnea, cough, attenuated breathing noise, foaming sputum | No | Weakness, dizziness | NA |
|  | Female, 91 years,  AB NA  Exacerbated COPD,  tuberculosis, asbestosis,  pleuritis, congestive heart failure | GGO, CP, UP, FS, SL, peripheral and central | 10 days | No | No | Dyspnea, foaming sputum | NA | Weakness, arthralgia | NA |
| ***(Continues)*** | | | | | | | | | |
| **Table S3 *(Continued)*** Characteristics of participants diagnosed COVID-19 positive by means of chest CT and/or RT-PCR and/or AB-test | | | | | | | | | |
| **Positive diagnostic test** | **Participant** | **Chest CT findings** | **Symptom onset before admission** | **Contact and/or association to cluster** | **Fever ^a^**  **(≥ 37.5 C°)** | **Respiratory symptoms** | **Gastro-**  **intestinal symptoms** | **General**  **symptoms** | **Oxygen satu-ration** |
|  | Male, 60 years,  AB neg  Metastatic colorectal  cancer, pericarditis | GGO, UP, N, peripheral and central | 5 days | No | No | Dyspnea, cough, attenuated breathing noise, foaming sputum | No | Weakness, dizziness | 94% |
|  | Female, 91 years,  AB NA  Exacerbated COPD,  tuberculosis, asbestosis,  pleuritis, congestive heart failure | GGO, CP, UP, FS, SL, peripheral and central | 10 days | No | No | Dyspnea, foaming sputum | NA | Weakness, arthralgia | 89% |
|  | Female, 86 years,  AB neg  Congestive heart failure,  pericarditis | GGO, UP, SL, peripheral | 12 days | No | No | Dyspnea, ambient breathing noise | No | No | 93% |
| AB, antibody test; COPD, chronic pulmonary obstructive disease; CP, crazy paving; CT, low-dose chest computed tomography; FS, fibrous strips; GGO, ground-glass-opacity; NA, not available; N, nodules; RT-PCR, reverse transcription polymerase chain reaction test; SL, subpleural lines; UP, unspecific patterns.  **^a^** tympanic or forehead temperature. | | | | | | | | | |

| **Table S4.** Laboratory findings of participants diagnosed COVID-19 positive by means of chest CT and/or RT-PCR and/or AB-test | | | | | | | | | | |
| --- | --- | --- | --- | --- | --- | --- | --- | --- | --- | --- |
| **Positive diagnostic test** | **Participant** | **Leucocyte count, x 10^9^ per L** | **Lymphocyte count, x 10^9^ per L** | **C-reactive protein, mg/L** | **Procalci-tonin, ng/mL** | **D-dimers, mg/L** | **Fibrino-gen, g/L** | **hs-cTnI,**  **pg/mL** | **NT-proBNP, pg/mL** | **Serum Ferritin, μg/L** |
| CT, PCR, AB | Male, 61 years  Congestive heart failure | 14.2 | NA | 8.7 | 0.1 | 88 | 5.4 | 2.9 | NA | 302 |
|  | Female, 76 years | 5.9 | 0.8 | 4.9 | NA | NA | 2.8 | NA | NA | NA |
|  | Male, 76 years  Congestive heart failure | 7.4 | 1.5 | 126 | 0.1 | 497 | 5.7 | 7.1 | NA | 266 |
| CT, PCR | Female, 65 years,  AB NA | 10.4 | 0.7 | 254 | 0.1 | 310 | 6.4 | 6.7 | NA | 1315 |
|  | Male, 69 years,  AB NA | 6.3 | 2.2 | 19 | NA | NA | NA | NA | NA | NA |
| CT, AB | Male, 70 years | 18.2 | 3.6 | 172 | 1.6 | 878 | 5.4 | 9.0 | NA | 172 |
|  | Male, 81 years  Congestive heart failure | 14.4 | NA | 228 | 1.9 | NA | NA | NA | NA | NA |
|  | Male, 82 years  Congestive heart failure | 38.8 | NA | 2.0 | NA | 778 | 2.3 | 12.3 | 709 | NA |
|  | Male, 86 years  Congestive heart failure | 22.5 | 1.3 | 146 | NA | NA | NA | NA | NA | NA |
| ***(Continues)***  **Table S4. *(Continued)*** Laboratory findings of participants diagnosed COVID-19 positive by means of chest CT and/or RT-PCR and/or AB-test | | | | | | | | | | |
| **Positive diagnostic test** | **Participant** | **Leucocyte count, x 10^9^ per L** | **Lymphocyte count, x 10^9^ per L** | **C-reactive protein, mg/L** | **Procalci-tonin, ng/mL** | **D-dimers, mg/L** | **Fibrino-gen, g/L** | **hs-cTnI,**  **pg/mL** | **NT-proBNP, pg/mL** | **Serum Ferritin, μg/L** |
|  | Male, 85 years | 11.3 | 1.6 | 137 | 0.1 | NA | NA | 23.6 | 317 | 114 |
|  | Male, 48 years | 9.6 | 0.7 | 74 | NA | NA | NA | NA | NA | NA |
| AB | Male, 60 years | 11.6 | 1.4 | 21 | 0.1 | NA | NA | 79.0 | 35 | NA |
|  | Female, 83 years | 3.6 | NA | 12 | NA | NA | NA | NA | 79 | 1527 |
| CT  (false positive) | Male, 58 years,  AB NA | 5.9 | 1.6 | 14 | 1.1 | 609 | 5.0 | 999 | NA | 47 |
|  | Female, 86 years,  AB NA  Congestive heart failure,  exacerbating COPD | 6.7 | 1.2 | 33 | 0.1 | 133 | 2.8 | 5.1 | NA | 135 |
|  | Male, 70 years,  AB neg  Congestive heart failure | 13.3 | 0.6 | 39 | 0.2 | 195 | 4.0 | 21.5 | NA | 154 |
| CT  (false positive) | Female, 76 years,  AB neg  Congestive heart failure | 11.0 | NA | 2.1 | 0.1 | NA | NA | 9.2 | 374 | NA |
|  | Male, 72 years,  AB neg | 9.5 | NA | 22 | NA | 4481 | NA | 32.1 | NA | NA |
| ***(Continues)*** | | | | | | | | | | |
| **Table S4. *(Continued)*** Laboratory findings of participants diagnosed COVID-19 positive by means of chest CT and/or RT-PCR and/or AB-test | | | | | | | | | | |
| **Positive diagnostic test** | **Participant** | **Leucocyte count, x 10^9^ per L** | **Lymphocyte count, x 10^9^ per L** | **C-reactive protein, mg/L** | **Procalci-tonin, ng/mL** | **D-dimers, mg/L** | **Fibrino-gen, g/L** | **hs-cTnI,**  **pg/mL** | **NT-proBNP, pg/mL** | **Serum Ferritin, μg/L** |
|  | Male, 60 years,  AB neg  Metastatic colorectal  cancer, pericarditis | 3.3 | NA | 96 | 0.7 | NA | NA | NA | 496 | 1488 |
|  | Female, 91 years,  AB NA  Exacerbated COPD,  tuberculosis, asbestosis, pleuritis,  congestive heart failure | 7.9 | NA | 24 | NA | NA | 3.9 | NA | NA | NA |
|  | Female, 86 years,  AB neg  congestive heart failure, pericarditis | 5.4 | 1.0 | 54 | 0.5 | 433 | 3.6 | 34.6 | NA | 4 |
| AB = antibody test; COPD = chronic pulmonary obstructive disease; CT = low-dose chest computed tomography; hs-cTnI = high-sensitivity cardiac troponin I; NA = not available; RT-PCR = reverse transcription polymerase chain reaction test. | | | | | | | | | | |


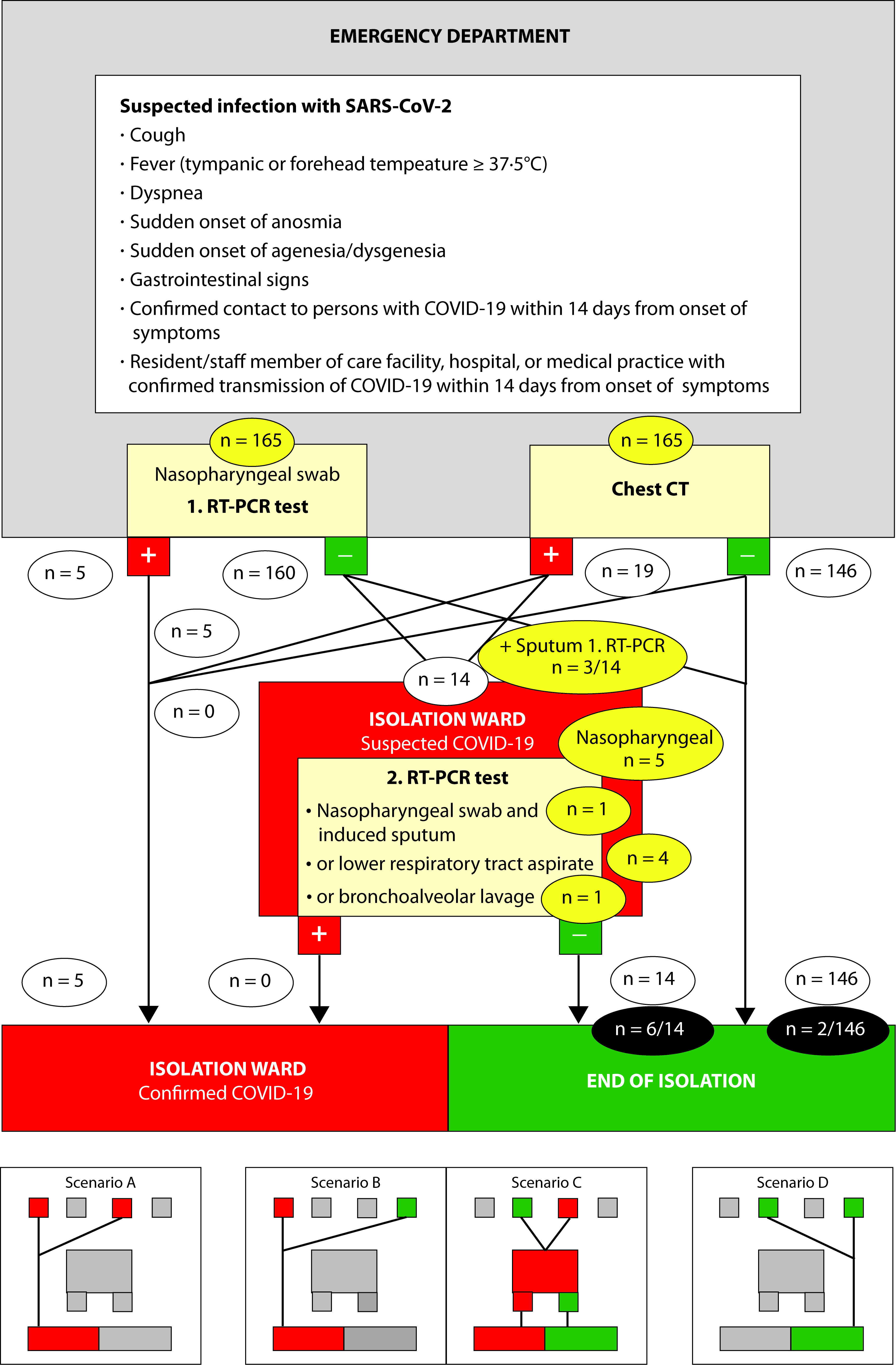


**Fig. S1** In-hospital patient flow chart including number of participants. Ellipses indicate number of participants with regard to conducted index/reference standard tests (yellow), results (white), and proportion of participants with false negative results (determined by positive antibody test from 3 weeks after symptom onset) (black). COVID-19, coronavirus disease 2019; CT, low-dose chest computed tomography; RT-PCR, reverse transcription polymerase chain reaction


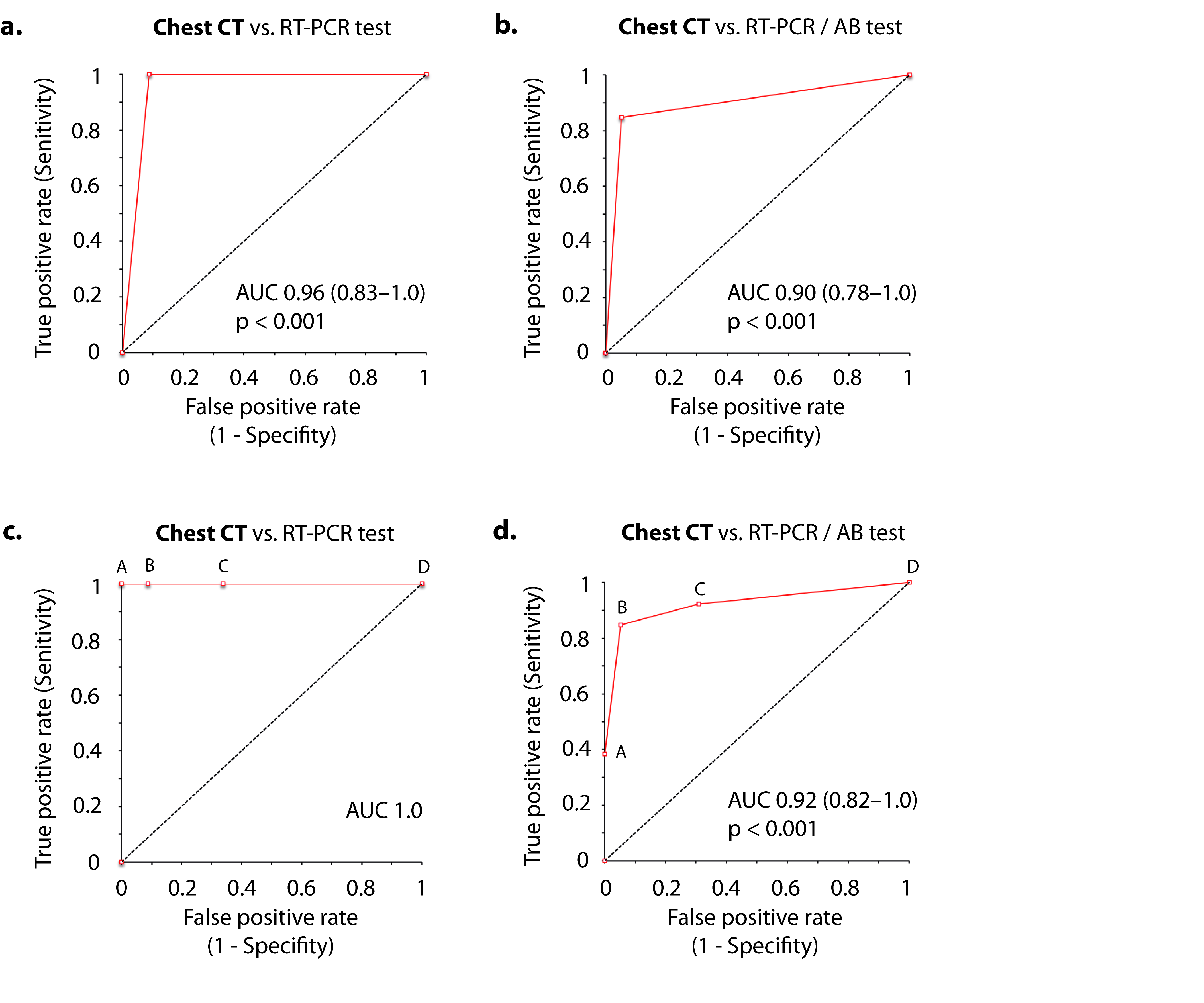


**Fig. S2** Receiver operating curves and areas under curves showing performance of chest CT for detection of COVID-19. **a** and **c** Receiver operating curve (ROC) and area under curve (AUC [95% CI) are given for the reference standard of RT-PCR alone, and **b** and **d** for the reference standard of RT-PCR and/or AB test. **a** and **b** Curves refer to the dichotomous outcome of COVID-19-positive or -negative participants, and **c** and **d** to the four-stage COVID-19 pneumonia imaging classification of typical appearance (A), nonspecific appearance (B), atypical appearance (C), and negative (D).

AB, antibody test; CT, computed tomography; RT-PCR, reverse transcription polymerase chain reaction.


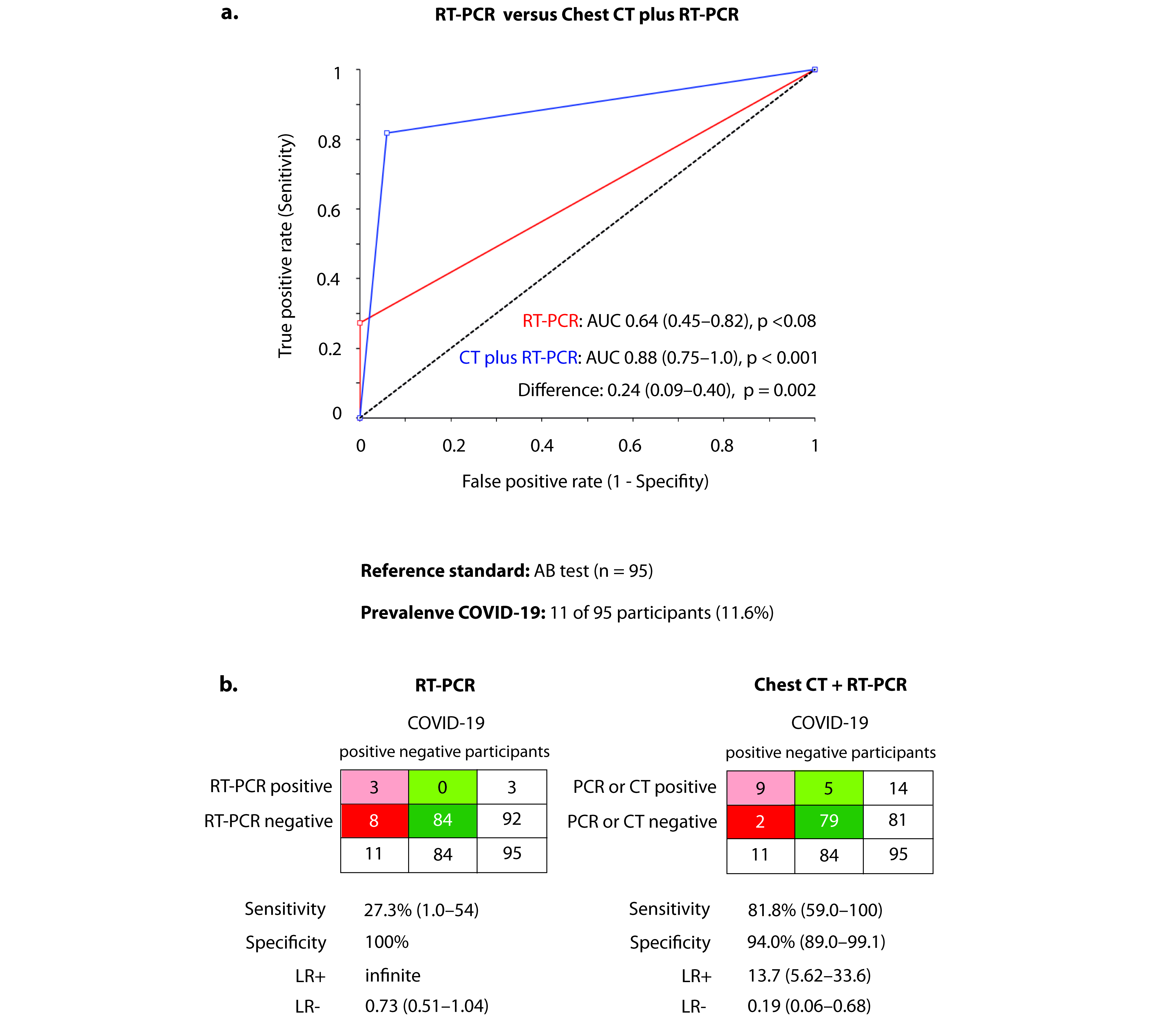


**Fig. S3** Comparison of diagnostic performance of RT-PCR alone with RT-PCR complemented by chest CT. Participants were considered COVID positive if either RT-PCR or chest-CT or both were positive. **a** Area under receiver operating curves (AUROCs) and **b** contingency tables show diagnostic performance based on the reference standard of antibody test. Test characteristics are presented with 95% confidence intervals (Wald-type). AB, antibody test; COVID-19, coronavirus disease 2019; CT, low-dose chest computed tomography; LR+, positive likelihood ratio; LR-, negative likelihood ratio; RT-PCR, reverse transcription polymerase chain reaction.


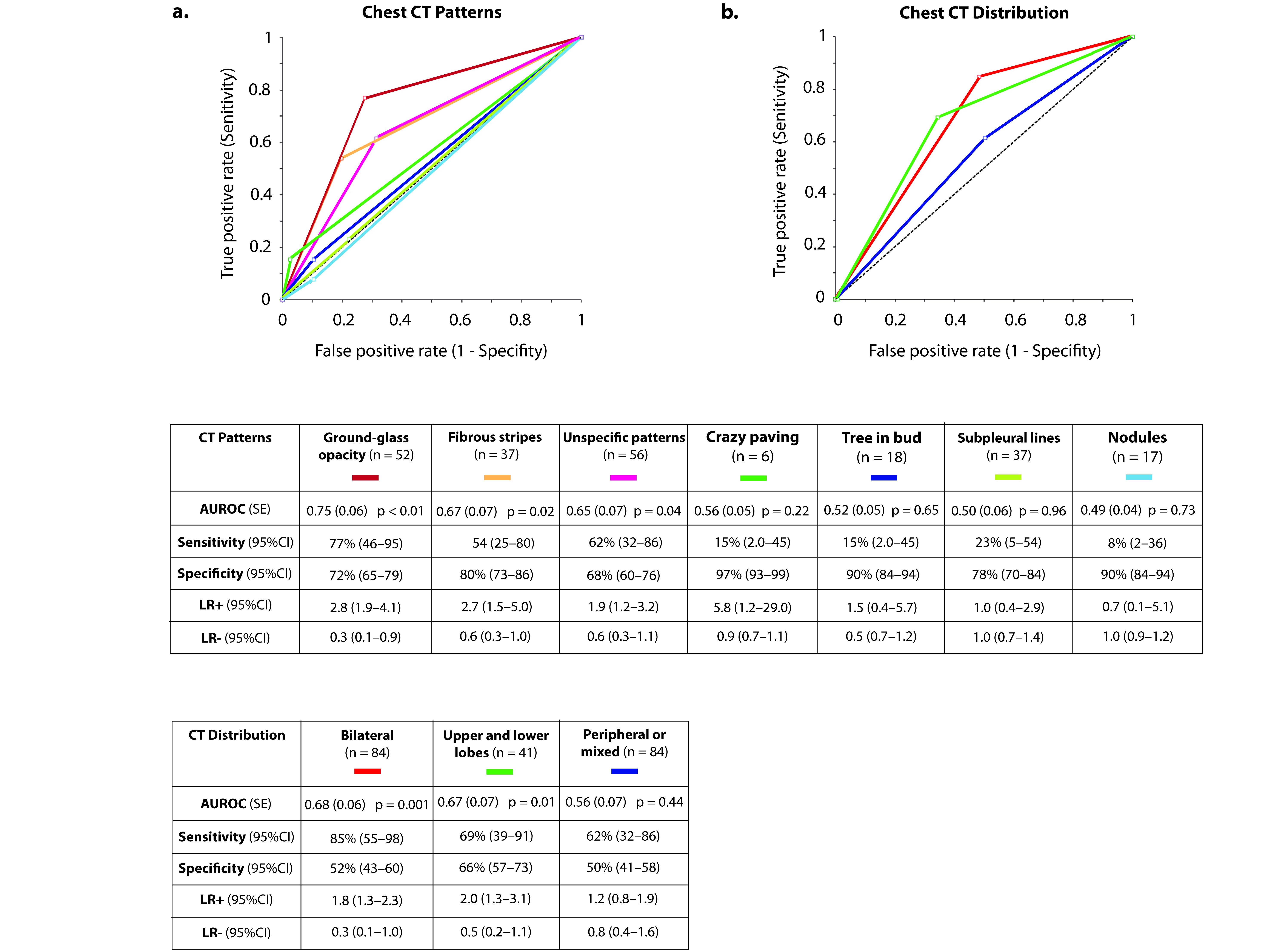


**Fig. S4** Areas under receiver operating curves showing distributing power of specific CT findings to detect COVID-19. **a** Areas under receiver operating curve (AUROC) are presented separately for patterns and **b** distributions of pathological signs. RT-PCR test- or, were available, AB test were considered as reference standard. CT, computed tomography, LR+, positive likelihood ratio; LR-, negative likelihood ratio

**
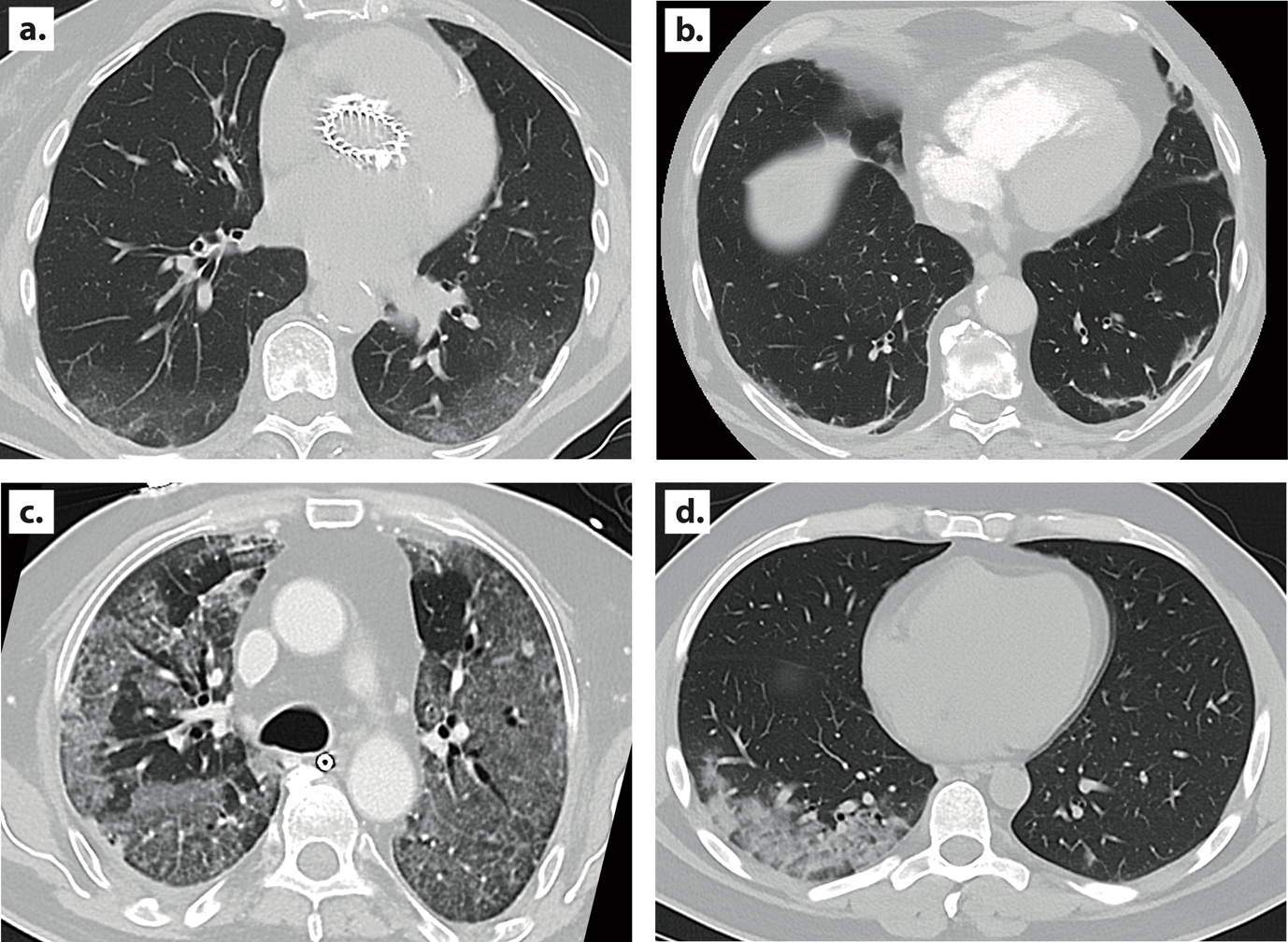
**

**Fig. S5** Thin-section axial CT images showing lungs of four adults with COVID-19. **a** 88-year-old female presenting with fever 7 days after transcatheter aortic valve implantation, CT shows bilateral, peripheral, focal areas of alveolar attenuation (ground-glass opacity). **b** 66-year-old male presenting with acute dyspnea and cough, CT shows bilateral, peripheral fibrous strips (interlobular septal thickening). **c** 68-year-old male presenting with acute respiratory distress syndrome after COVID-19 pneumonia, CT shows diffuse bilateral opacities with crazy paving pattern (intralobular lines), subpleural lines, and interstitial septal thickening. **d** 43-year-old male presenting with dyspnea, CT shows alveolar infiltration (unspecific pattern).

**References**

1. Baeyens JP, Serrien B, Goossens M, Clijsen R. Questioning the "SPIN and SNOUT" rule in clinical testing. *Arch Physiother* 2019;9:4.

2. Corman VM, Landt O, Kaiser M, et al. Detection of 2019 novel coronavirus (2019-nCoV) by real-time RT-PCR. *Euro Surveill* 2020; 25(3).

3. Krüttgen A, Cornelissen CG, Dreher M, Hornef M, Imöhl M, Kleines M. Comparison of four new commercial serologic assays for determination of SARS-CoV-2 IgG. *J Clin Virol* 2020;128:104394.

4. Simpson S, Kay FU, Abbara S, et al. Radiological Society of North America Expert Consensus Statement on Reporting Chest CT Findings Related to COVID-19. Endorsed by the Society of Thoracic Radiology, the American College of Radiology, and RSNA - Secondary Publication. *J Thorac Imaging.* 2020;35(4):219-227.
